# The Way of Transgressors

**Published:** 1881-01

**Authors:** 


### THE WAY OF TRANSGRESSORS.

Last week, in the case of Dr. John Buchanan, formerly Dean of the Eclectic Medical College, Pine Street, above Fourth, Philadelphia, and of the American University of Pennsylvania, charged with conspiracy to defraud the United States, a fine of $500 was imposed by Judge Butler, in the United States District Court. Added to this were the costs of the prosecution, and the prisoner will also have to undergo ten months' imprisonment. M. V. Chapman, in the same case was fined $500 and costs and sentenced to a year and ten months' imprisonment.

